# Breakage and migration of a high-speed dental hand-piece bur during mandibular third molar extraction: Two case reports

**DOI:** 10.1097/MD.0000000000019177

**Published:** 2020-02-14

**Authors:** Shinpei Matsuda, Hitoshi Yoshimura, Hisato Yoshida, Kazuo Sano

**Affiliations:** a Department of Dentistry and Oral Surgery, Unit of Sensory and Locomotor Medicine, Division of Medicine, Faculty of Medical Sciences, University of Fukui; b Oral Care Support Center, Fukui Dental Association, Fukui, Japan.

**Keywords:** high-speed dental hand-piece bur, iatrogenic foreign body, mandibular third molar extraction

## Abstract

**Rationale:**

Tooth extraction is a common dental surgical procedure. There is a possibility that various complications often occur during third molar tooth extractions.

**Patient concerns:**

The authors report herein 2 cases of migration of a high-speed dental hand-piece bur during mandibular third molar extraction—one case with the iatrogenic foreign body migrating into the mandibular body and another case with the iatrogenic foreign body migrating into the floor of mouth are reported.

**Diagnosis:**

The patient was diagnosed with the iatrogenic foreign body associated with mandibular third molar extraction by imaging examinations.

**Interventions:**

The authors performed elective surgery to remove the foreign body under general anesthesia in Case 1, and performed emergency surgery to remove the foreign body under local anesthesia in Case 2.

**Outcomes:**

The foreign bodies were removed, and complete removal of the foreign bodies was confirmed by postoperative x-ray examination. The patients’ postoperative courses were uneventful.

**Lessons:**

The selection of adequate surgical procedures and instruments will prevent the occurrence of iatrogenic foreign bodies. If migration accidents occur, their positions should first be confirmed by imaging examinations. Dentists and/or oral surgeons should perform removal operations considering the degree of emergency based on the results of imaging examinations.

## 1 Introduction

Medical or dental materials to be removed, such as gauzes and broken pieces of medical or dental devices, are usually detected during surgery, and surgeons should try to remove them. Additionally, if they are left in the body, the patients usually complain of some symptoms, such as swelling and pain associated with infection.^[1]^ Therefore, it is an uncommon and undesirable case that is associated with an iatrogenic foreign body being detected incidentally by imaging examinations.^[1]^ Technological progress, such as dental cone beam computed tomography (CT), 3-dimensional (3D) navigation, and image-processing methods, has contributed to innovations in surgical procedures, thereby leading to better visualization of surgical fields and the lesions or objects to be removed not only in medical practices but also in dental practices.^[2,3]^ However, unfortunately, there is no end to cases associated with iatrogenic migration of foreign bodies, even today. Even in the oral and maxillofacial regions, there were no exceptions, and some research has reported iatrogenic migration of foreign bodies in these regions.^[1]^

Tooth extraction is a common dental surgical procedure. However, it is sometimes not easy, and there is a possibility that various complications associated with local anesthesia, infection, bleeding, damage to adjacent structures (including nerves), emphysema, and breakage and migration of dental instruments may occur during tooth extraction.^[4–8]^ They often occur, especially during third molar tooth extractions. Our previous study clarified that high-speed dental hand-pieces, such as the air-driven hand-piece and electrical hand-piece, could cause pneumomediastinum during third molar tooth extractions.^[5]^ As a result, prudence and certainty are required for dentists and oral surgeons in every step of tooth extractions in all of the sites.

There have been few reports about migration of a high-speed dental hand-piece bur during mandibular third molar extraction.^[6–8]^ The authors reported 2 cases of migration of a high-speed dental hand-piece bur during mandibular third molar extraction.

### 1.1 Consent

Written informed consent was obtained from the patient for publication of the case and any accompanying images.

## 2 Case report

### 2.1 Case 1

**Figure 1:**
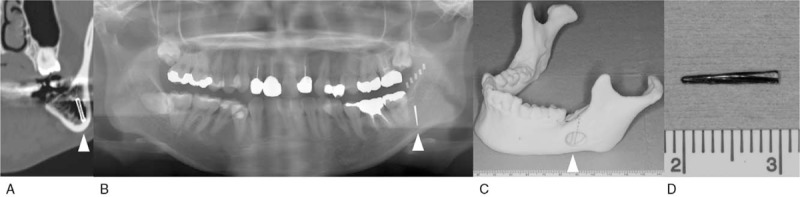
Case 1. (A) Preoperative computed tomography. (B) Preoperative panoramic radiograph with a stent that enclosed the radiopaque materials. (C) Three-dimensional model created based on the preoperative computed tomography. (D) The foreign body.

A 36-year-old woman visited a dental clinic for dental treatment, and a foreign body in the left mandible was incidentally detected on panoramic radiograph. The patient was referred to our department for removal of the foreign body in the left mandible. The patient had no remarkable medical or trauma history, but she received left impacted mandibular third molar extraction 8 years ago. Physical and intraoral examination did not show abnormal findings. Preoperative CT examination revealed a straight radiopaque foreign body having a length of 10 mm in the left mandibular body (Fig. 1A). The authors planned elective surgery to remove the foreign body under general anesthesia in consideration of the absence of subjective symptoms, the long time that has passed since migration of the foreign body, and the low risk of damage to other organs because the foreign body remained in the mandibular bone. A panoramic radiograph was obtained with a stent that enclosed the radiopaque material to determine the migration position (Fig. 1B), and a 3D model was created based on the preoperative CT image (Fig. 1C). They were used for making a surgical plan to minimize healthy bone damage. The authors reached the foreign body by elevating the mucoperiosteal flap and removing the alveolar crest bone of the right mandibular third molar region. Then, the foreign body was removed, and complete removal of the foreign body was confirmed by intraoperative x-ray examination. The foreign body was a piece of dental hand-piece bur, and it was thought that the bur migrated during third molar extraction (Fig. 1D). The postoperative course was uneventful.

### 2.2 Case 2

**Figure 2:**
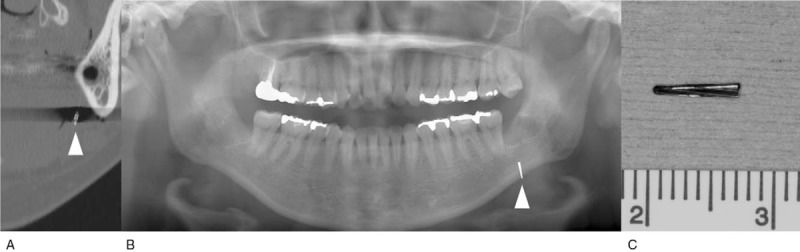
Case 2. (A) Preoperative computed tomography. (B) Preoperative panoramic radiograph. (C) The foreign body.

A 33-year-old woman visited a dental clinic for left mandibular third molar extraction. Because the dental hand-piece bur broke and migrated into the floor of mouth during tooth extraction, the patient presented to our department for removal of the fractured bur fragment on that day. Physical examination showed no remarkable symptoms. Intraoral examination showed an extraction wound of the mandibular left third molar, and the fractured bur fragment could not be confirmed. Preoperative CT examination revealed a straight radiopaque foreign body having a length of 7 mm beneath the mucous membrane of the mouth's floor (Fig. 2A, B). The authors performed emergency surgery to remove the foreign body under local anesthesia in consideration of the risk of removal and damage to other organs, because the foreign body remained in the soft tissue. A careful search using a fiber light accompanied by suctioning and compression to the submandibular region contributed to the detection of the dental hand-piece bur. The foreign body was removed, and complete removal of the foreign body was confirmed by postoperative x-ray examination (Fig. 2C). The postoperative course was uneventful.

It was considered that the high-speed dental hand-piece burs associated with case 1 and case 2 were the same burs made of steel with tungsten carbide from these forms.

## 3 Discussion

In both cases presented in this report, the pieces of the dental hand-piece burs associated with the third molar extraction had migrated. However, they were different in the detected situation, in the migration position, and in the period between migration and removal. The authors considered that comparison of these cases in this report provides important information for dentists and oral surgeons in selecting removal methods of foreign bodies, including the timing of removal surgery under local or general anesthesia. Bouloux et al^[4]^ discussed the displacement of the mandibular third molar, and concluded that delayed intervention for the displaced tooth might carry the risk of infection, thrombosis, erosion into the carotid artery or one of its branches, and interference with some cranial nerves. These undesirable events may also occur as a result of the migration of foreign bodies into soft tissues. Therefore, foreign bodies that have migrated into soft tissues must be removed as soon as possible. On the contrary, in case 1, the foreign body migrated into the mandibular bone and did not cause any symptoms for a long time. The 2 cases in this report suggested that the migrated position of the foreign body influenced the degree of emergency. If elective surgery can be selected, imaging examinations with stents enclosing the radiopaque materials and 3D models could provide useful information for dentists and oral surgeons.

Iatrogenic foreign bodies are usually associated with inadequate technique or inadequate selection of an instrument.^[1]^ High-speed dental hand-piece burs have high cutting efficiency, and dentists should always pay attention to the probability of causing overheating, emphysema, and/or breakage.^[5,6]^ Additionally, thin high-speed dental hand-piece burs made of steel with tungsten carbide or diamond coatings are not designed for the removal of hard cortical bone.^[8]^ Interestingly, high-speed dental hand-piece burs were broken at different points in this report, although the forms of the foreign bodies were very similar and the mandibular third molar extractions were on the same side. This result suggested that the application of force during cutting has a greater effect on breakage than the original physical characteristics of the dental high-speed burs. It was highly possible that bur breakages were caused by application of inadequate force and/or inadequate instruments in both cases presented in this report. The observation of basic rules of surgical procedures may help to avoid these kinds of iatrogenic accidents.^[1]^

If iatrogenic migration occurs, the dentists or oral surgeons may try to remove the foreign bodies. However, especially in cases of foreign bodies in soft tissues, blind surgical exploration affected by blood and saliva may make a bad situation worse. In case 2, a fiber light accompanied by suctioning and compression to the submandibular region contributed to the detection of the foreign body associated with third molar extraction. Adequate medical devices and the calmness of all staff will lead to the solutions of these problems.

If the foreign bodies were constructed of wood or bamboo, then image-processing software, such as OsiriX, might be useful for distinguishing those from other important structures.^[2]^ On the contrary, image-processing technology could not be used because the influence of the metallic artifact in CT could not be removed in this study. A real-time 3D navigation system might only be useful in elective surgery under general anesthesia, because the unpredictable movements of patients affect the accuracy of the navigation image.^[3,9]^

In conclusion, the selection of adequate surgical procedures and instruments will prevent the occurrence of iatrogenic foreign bodies. If migration accidents occur, their positions should first be confirmed by imaging examinations. Dentists and/or oral surgeons should perform removal operations considering the degree of emergency based on the results of imaging examinations.

## Author contributions

**Conceptualization:** Shinpei Matsuda.

**Writing – original draft:** Shinpei Matsuda.

**Writing – review & editing:** Shinpei Matsuda, Hitoshi Yoshimura, Hisato Yoshida, Kazuo Sano.

## References

[1] Matsuda S Ohba S Yoshimura H Assessment of migrated foreign bodies in the maxillae by x-ray fluorescence spectrometry. J Craniofac Surg 2014;25:e233–5. 24777003 10.1097/SCS.0000000000000515

[2] Matsuda S Yoshimura H Yoshida H Usefulness of computed tomography image processing by OsiriX Software in detecting wooden and bamboo foreign bodies. Biomed Res Int 2017;3104018. 29119104 10.1155/2017/3104018 PMC5651097

[3] Matsuda S Yoshimura H Yoshida H Application of a real-time three-dimensional navigation system to dental implant removal: a five-year single-institution experience. J Hard Tissue Biol 2018;27:359–62.

[4] Bouloux GF Steed MB Perciaccante VJ Complications of third molar surgery. Oral Maxillofac Surg Clin North Am 2007;19:117–28. 18088870 10.1016/j.coms.2006.11.013

[5] Ohta K Yoshimura H Ryoke T Investigation of the electric handpiece-related pneumomediastinum and cervicofacial subcutaneous emphysema in third molar surgery. J Hard Tissue Biol 2019;28:79–86.

[6] Yalcin S Aktas I Emes Y Accidental displacement of a high-speed handpiece bur during mandibular third molar surgery: a case report. Oral Surg Oral Med Oral Pathol Oral Radiol Endod 2008;105:e29–31. 10.1016/j.tripleo.2007.09.017 18280942

[7] Ali FM Khan MA Shtaifi AE Accidental high-speed hand piece bur buried during surgery of mandibular third molar: a rare case report. MOJ Clin Med Case Rep 2016;4:152–3.

[8] Rajaran JR Nazimi AJ Rajandram RK Iatrogenic displacement of high-speed bur during third molar removal. BMJ Case Rep 2017;2017. 10.1136/bcr-2017-221892 PMC5747789 28954756

[9] Matsuda S Yoshimura H Sano K Application of a real-Time 3-dimensional navigation system for treatment of synovial chondromatosis of the temporomandibular joint: a case report. Medicine (Baltimore) 2019;98:e15382. 31045787 10.1097/MD.0000000000015382 PMC6504332

